# Mesmerism in Holland

**Published:** 1849-10

**Authors:** 


*Mesmerism in Holland.*—The supreme court of the Netherlands has just given a verdict which runs pretty nearly as follows:—"A magnetizer who employs a person, who, during sleep, points out remedies to the patients who come for advice, exercises medicine illegally, when unprovided with a diploma." "It is no excuse for him to be paying for a license as a magnetizer." The courts of Liege and Brussels have done more still; they have found a verdict against a magnetizer for administering magnetized water.—*Belgique Judiciare.*

